# Safety and Efficacy of Mesenchymal Stem Cell Therapy in Aging Frailty: A Systematic Review

**DOI:** 10.3390/ijms27177596

**Published:** 2026-08-25

**Authors:** Eleni Poutouri, Maria Sotiropoulou, Michael Potoupnis, Georgios Koliakos, Evanthia Kassi, Symeon Tournis, Panagiotis Anagnostis

**Affiliations:** 1 MSc Program “Stem Cells and Regenerative Medicine”, School of Medicine, Aristotle University of Thessaloniki, 54124 Thessaloniki, Greece; 2 Academic Orthopedic Department, “Papageorgiou” General Hospital, School of Medicine, Aristotle University of Thessaloniki, 54124 Thessaloniki, Greece; 3 Centre of Expertise in Rare Endocrine Diseases, C.E.R.E.D—Disorders of Calcium and Phosphate Metabolism, Endocrinology Unit, 1st Department of Propaedeutic Internal Medicine, LAIKO General Hospital of Athens, National and Kapodistrian University of Athens, 10679 Athens, Greece; 4 Laboratory for Research of the Musculoskeletal System “Th. Garofalidis”, Medical School, National and Kapodistrian University of Athens, KAT Hospital, 14561 Athens, Greece; 5 Division of Endocrinology, 3rd Department of Internal Medicine, “Papageorgiou” General Hospital of Thessaloniki, School of Medicine, Aristotle University of Thessaloniki, 54124 Thessaloniki, Greece

**Keywords:** aging frailty, mesenchymal stem cells, systematic review, regenerative medicine, physical function

## Abstract

Aging frailty is a multifactorial geriatric syndrome characterized by reduced physiological reserve across multiple interrelated systems, leading to increased vulnerability to adverse health outcomes. Mesenchymal stem cells (MSCs) have emerged as a potential therapeutic intervention due to their immunomodulatory and regenerative properties. This systematic review aimed to evaluate the available clinical evidence regarding the safety and efficacy of MSC administration in individuals with aging frailty. A systematic literature search was conducted in PubMed, Scopus, and the Cochrane Library from database inception to 15 April 2026. Only randomized controlled trials (RCTs) were eligible. Data were extracted according to study design, participants’ characteristics, MSC source and dosing, safety outcomes, functional performance measures, quality of life (QoL) indices, and inflammatory, immune, and vascular biomarkers. Three RCTs met the inclusion criteria, comprising a total of approximately 208 participants. MSC administration was well-tolerated, with no treatment-related serious adverse events reported. Signals of improvement were reported in mobility-related and physical performance outcomes, although the magnitude and consistency of the response varied across studies. A dose-dependent increase in 6 min walk distance was observed at nine months in one trial, while improvements in SPPB, TUG, and grip strength were reported in the other studies. Additionally, MSC therapy was associated with QoL improvement and reduction in circulating pro-inflammatory cytokine concentrations. MSC administration was also associated with changes in immune-cell activation and a dose-dependent reduction in circulating sTIE2. Because of substantial clinical and methodological heterogeneity among the included trials, a quantitative meta-analysis was not performed. Current randomized evidence remains limited but suggests that intravenous MSC therapy is generally well tolerated in older adults with aging frailty and may produce clinically relevant signals of improvement in mobility-related functional outcomes, quality of life, and selected inflammatory, immune, and vascular biomarkers. However, the small number of trials, heterogeneity in MSC source, dose, endpoints, and follow-up duration, and the exploratory nature of much of the evidence preclude definitive conclusions regarding efficacy. Larger, adequately powered trials with standardized frailty endpoints are required, while future trials specifically targeting sarcopenia should apply consensus diagnostic criteria and direct measures of muscle quantity, strength, and physical performance.

## 1. Introduction

Age-related frailty is a multidimensional geriatric syndrome characterized by reduced reserve and function across multiple physiological systems and by increased vulnerability to stressors, resulting in a disproportionate risk of adverse health outcomes [1]. The Fried physical frailty phenotype operationalizes frailty as the presence of at least three of five criteria: unintentional weight loss, self-reported exhaustion, weakness, slow walking speed, and low physical activity; the presence of one or two criteria indicates prefrailty [2]. In contrast, the Clinical Frailty Scale provides a clinician-rated global assessment that incorporates mobility, functional independence, cognition, and comorbidity [3]. Sarcopenia is a related but distinct progressive and generalized skeletal muscle disorder. According to the revised European Working Group on Sarcopenia in Older People consensus, low muscle strength indicates probable sarcopenia, low muscle quantity or quality confirms the diagnosis, and the additional presence of impaired physical performance indicates severe sarcopenia [4]. Disability is also a distinct construct and, in older adults, generally refers to difficulty or dependence in performing activities required for independent living [5]. Although sarcopenia may contribute to physical frailty and both conditions may increase the risk of disability, frailty, sarcopenia, and disability should not be used interchangeably [4,5].

Frailty and sarcopenia both become more common with advancing age, although epidemiological estimates are strongly influenced by the population studied, the care setting, and the operational definition applied [6,7,8]. In an age-stratified meta-analysis of community-dwelling older adults in China, the pooled prevalence of frailty increased from approximately 6% among individuals aged 65–74 years to 15% among those aged 75–84 years and 25% among those aged ≥ 85 years [9]. Among participants in the US-based 90+ Study, assessed using a physical frailty phenotype, prevalence was 24.0% at 90–94 years and 39.5% at ≥95 years [10]. Regarding incidence, a global systematic review and meta-analysis of community-dwelling adults aged ≥ 60 years estimated 43.4 new cases of frailty (95% CI: 37.3–50.4) and 150.6 new cases of prefrailty (95% CI: 123.3–184.1) per 1000 person-years [11]. This steep age gradient should not, however, be interpreted as indicating that frailty is an inevitable or clinically normal consequence of advanced age. A substantial proportion of individuals remain non-frail even at very advanced ages; therefore, clinically meaningful frailty should be identified using validated criteria demonstrating diminished physiological reserve and disproportionate vulnerability to adverse outcomes rather than chronological age alone [1,4,6].

The prevalence of sarcopenia also increases markedly with age, but estimates vary substantially according to the diagnostic framework, component-specific thresholds, reference population, and measurement protocols. In two Swedish population-based cohorts, sarcopenia prevalence ranged from 1.4% to 7.8% at 70 years and from 42% to 62% at 85 years, depending on the EWGSOP definition and cut-off values applied [7]. In the Newcastle 85+ Study, in which sarcopenia was defined according to the original EWGSOP criteria, prevalence was 21% at a mean age of 85.5 years, while the incidence rate was 3.7 cases per 100 person-years [8]. Consequently, comparisons of sarcopenia prevalence or incidence across studies require explicit reporting of the diagnostic consensus used, the thresholds for muscle strength and muscle quantity, the method used to assess muscle mass, and the physical-performance protocol [4,7,8].

An accumulating body of evidence indicates that frailty is closely linked to biological processes associated with aging, including chronic low-grade inflammation, immune dysregulation, mitochondrial dysfunction, and impaired tissue repair capacity [4]. These mechanisms collectively contribute to functional decline and reduced resilience to physiological stressors [4,12,13]. In this context, mesenchymal stem cells (MSCs) have attracted considerable attention as a potential therapeutic approach due to their ability to modulate immune responses, attenuate systemic inflammation, and promote tissue homeostasis through paracrine signaling [14,15,16,17]. At the cellular and molecular level, MSC therapy may influence several processes involved in aging frailty, particularly chronic immune activation and endothelial dysfunction. The available clinical studies have reported changes in circulating TNF-α and IL-17 concentrations, activated T-cell populations, B-cell intracellular TNF-α, and soluble TIE2 (sTIE2), a circulating form of the endothelial TIE2 receptor [18,19,20]. These findings suggest that the effects of MSCs may be mediated, at least in part, through immunomodulatory and paracrine mechanisms rather than direct tissue replacement. Early-phase clinical trials have suggested that MSC therapy may exert beneficial effects on physical performance and inflammatory status in frail older adults [19,20]. However, the clinical relevance, consistency, and durability of these effects remain uncertain. So far, no consensus exists regarding their safety profile, optimal cell source, dosing strategy, or therapeutic efficacy in aging frailty and sarcopenia.

Recent reviews have addressed MSC-based strategies in sarcopenia and osteosarcopenia, focusing on exercise-mediated MSC activation, the broad epidemiology and management of osteosarcopenia, or the preclinical biology and optimization of human umbilical-cord-derived MSCs [21,22,23]. However, none of these reviews restricted the evidence synthesis to randomized controlled trials of intravenous MSC administration in clinically defined aging frailty or incorporated the recently published phase 2b dose-escalation trial. The present review addresses this evidence gap by jointly evaluating safety and functional outcomes, MSC source, dose, manufacturing and potency characteristics, and inflammatory, immune, and vascular biomarkers.

The aim of the present study was to systematically review and critically appraise randomized controlled trials evaluating the safety and efficacy of intravenous MSC therapy in older adults with aging frailty and to determine whether any eligible trials specifically targeted consensus-defined sarcopenia.

## 2. Methods

### 2.1. Guidelines and Protocol Registration

This systematic review was conducted in accordance with the Preferred Reported Items for Systematic Review and Meta-Analyses (PRISMA) guidelines. A predefined protocol guided all stages of the review process, including literature search, study selection, data extraction, and data synthesis. The review protocol was registered in the PROSPERO database (registration number: CRD420251174715).

### 2.2. Search Strategy

A comprehensive literature search was performed in PubMed, Scopus, and the Cochrane Library from database inception to 15 April 2026. Search terms included controlled vocabulary and free-text keywords related to aging frailty, frailty syndrome, physical performance, MSCs, and stem cell therapy. Boolean operators were used to combine terms, and the search strategy was adapted to the syntax of each database. Reference lists of eligible articles were manually screened to identify additional relevant studies.

### 2.3. Study Selection

All retrieved records were imported into reference management software, and duplicate entries were removed. Two authors (EP and MS) independently screened titles and abstracts for eligibility. Full-text assessment was subsequently performed for potentially relevant studies. Eligibility decisions were made independently by both reviewers, with full agreement achieved at all stages. The following PICO (Population, Intervention, Comparison and Outcome) elements were set as inclusion criteria: (i) Population: patients with sarcopenia or frailty, (ii) Intervention: stem cells of any type, (iii) Comparison: placebo or usual care and (iv) Outcome: mean difference in muscle strength, mass and performance. Only randomized controlled trials (RCTs) were eligible. Exclusion criteria were: (i) non-RCTs, (ii) studies specifically conducted in patients with chronic diseases associated with sarcopenia or frailty, (iii) non-English papers and (iv) animal studies.

### 2.4. Data Extraction

Two independent researchers extracted data from eligible studies that meet the predefined criteria. A standardized form was used to record: (i) first author, (ii) year of publication, (iii) country in which the study was conducted, (iv) study duration, (v) total sample size, (vi) mean participant age and body mass index, (vii) number of patients in each group, (viii) dose and type of stem cells and (ix) mean difference (MD) in muscle mass, muscle strength and/or physical performance in each group. Where available, data on MSC phenotype, passage number, cell viability, manufacturing characteristics, potency assessment, and circulating inflammatory, immune, vascular, and angiogenesis-related biomarkers were also extracted.

### 2.5. Risk of Bias Assessment

The revised Cochrane Risk of Bias tool for randomized trials (RoB 2) was used to assess the risk of bias in the three included randomized controlled trials [24].

### 2.6. Data Analysis

Given the small number of eligible trials and the substantial clinical and methodological heterogeneity in population definitions, MSC source and dose, outcome measures, and follow-up time points, a quantitative meta-analysis was not considered methodologically appropriate. The findings were therefore synthesized narratively. Continuous outcomes were summarized using the effect estimates reported in the original trials, including within-group changes and between-group differences, together with 95% confidence intervals and *p* values where available. Safety outcomes were summarized descriptively. No new inferential analyses were performed.

## 3. Results

### 3.1. Characteristics of Studies Included for Analysis

**Figure 1:**
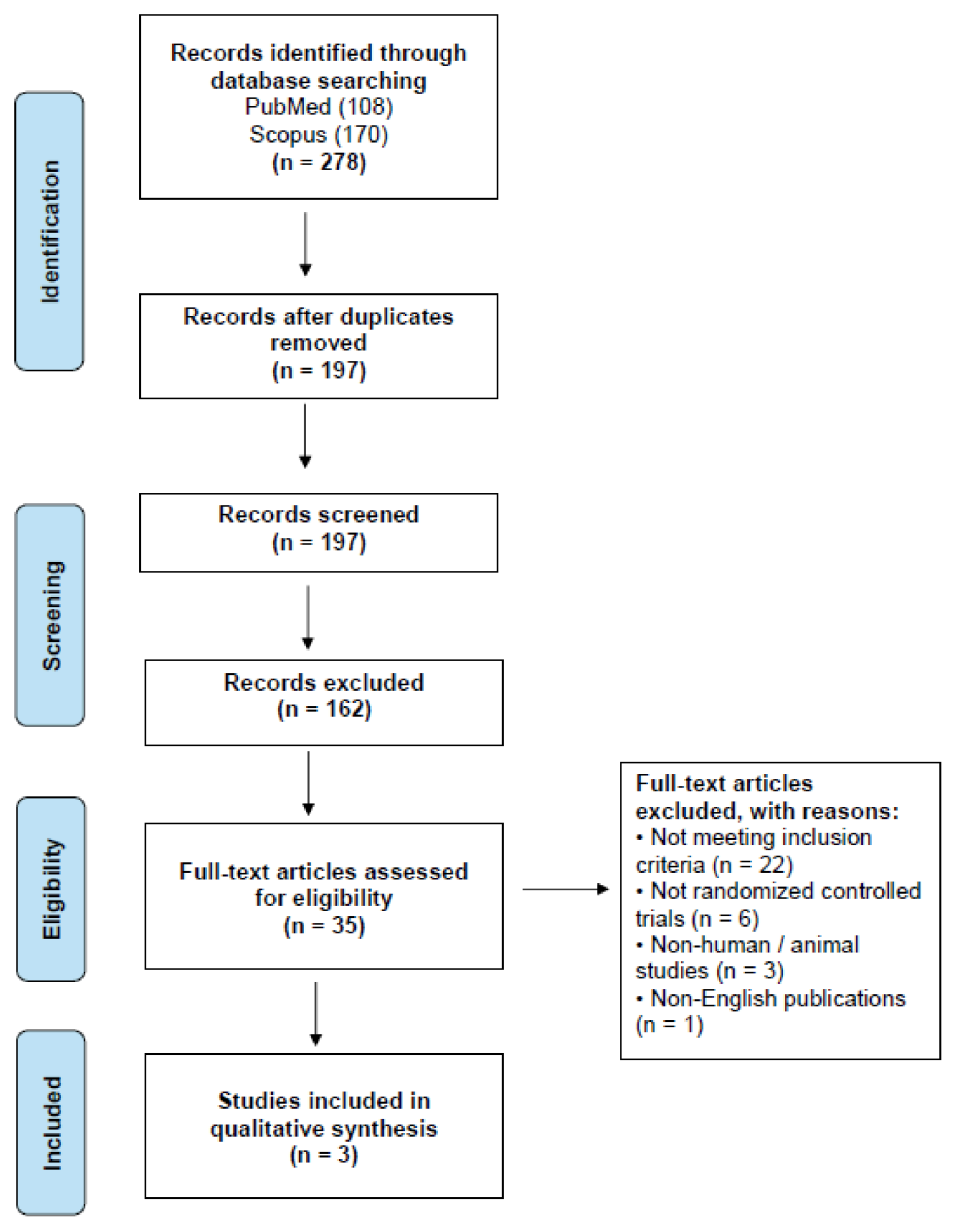
PRISMA flowchart diagram of the study selection process.

The initial search yielded 278 records across all databases. After removal of duplicates, 197 records remained. Following title and abstract screening, 35 studies were selected for full-text eligibility, 3 of which met the predefined inclusion criteria and were included in the qualitative synthesis [18,19,20]. Although sarcopenia constitutes a major biological substrate of frailty, no randomized controlled trials specifically targeting sarcopenia were identified. A flowchart of the study selection process is presented in Figure 1.

Overall, 208 participants with aging frailty were included, with study sample sizes ranging from 30 to 148 and mean ages from 67 to 85 years.

Tompkins et al. [19] conducted a phase II RCT in older adults with clinically defined frailty, evaluating the intravenous IV administration of allogeneic bone-marrow-derived MSCs at doses of 100 million and 200 million cells versus placebo [19]. The primary outcome of the study was safety and tolerability, while secondary outcomes included functional performance assessed by the 6 min walk test (6MWT), the short physical performance battery (SPPB), and pulmonary function measures such as forced expiratory volume in 1 s (FEV1). However, the study was not powered to detect definitive efficacy differences, and the divergent responses between dose groups suggest a potentially non-linear dose–response pattern that could not be formally explored [19].

In another randomized, double-blind, placebo-controlled trial, Zhu et al. [20] evaluated umbilical-cord-derived MSCs in frail older adults over a 6-month follow-up period. Functional performance was assessed using multiple endpoints, including timed up-and-go (TUG), the four-meter walking test (4MWT), and grip strength, alongside quality-of-life measures and circulating inflammatory biomarkers such as TNF-α and IL-17 [20]. Improvements were observed across several domains; nevertheless, the modest sample size and variability across endpoints limit the robustness and generalizability of these findings [20].

The largest and most methodologically rigorous study to date is the phase 2b randomized, double-blind, placebo-controlled, dose-escalation trial by Ruiz et al. [18], which included 148 participants, aged 70–85 years. Participants received a single IV infusion of allogeneic bone-marrow-derived MSCs (laromestrocel) at escalating doses (25, 50, 100, and 200 million cells) or placebo, with follow-up extending to nine months [18]. The 6MWT was predefined as the primary endpoint, whereas additional functional and patient-reported outcomes were assessed as secondary endpoints [18]. The larger sample size, together with the dose-escalation design, provides a substantially more robust framework for evaluating therapeutic efficacy compared with earlier trials [18]. The MSC products were not identical across the included trials. Tompkins et al. [19] and Ruiz et al. [18] administered allogeneic bone-marrow-derived MSCs, whereas Zhu et al. [20] used fifth-passage human umbilical-cord-derived MSCs manufactured under good manufacturing practice conditions. In the study by Zhu et al., the cells expressed CD73, CD90, and CD105, lacked CD11b, CD19, CD31, CD34, CD45, and HLA-DR, retained trilineage differentiation capacity, and had a viability above 90% [25]. Ruiz et al. additionally reported lot-specific potency testing using laromestrocel-conditioned medium. These differences in cell source, manufacturing, dose, and potency assessment should be considered when comparing biological and clinical responses across studies [18,20].

The operational definitions and severity of frailty differed substantially across the included trials. Tompkins et al. enrolled adults aged 60–95 years with physician-assessed Clinical Frailty Scale scores of 4–7. At baseline, 57% of participants had a score of 4, 30% had a score of 5, 13% had a score of 6, and none had a score of 7, indicating that the study population predominantly comprised vulnerable or mildly frail individuals [19]. Zhu et al. enrolled adults aged 60–80 years with Fried frailty phenotype scores of 1–4. The median baseline score was 2 in both study groups, indicating that the population included both prefrail and frail participants and was predominantly positioned toward the prefrailty range [20]. In contrast, Ruiz et al. applied more restrictive eligibility criteria, enrolling cognitively unimpaired adults aged 70–85 years with Clinical Frailty Scale scores of 5 or 6, a screening 6 min walk distance of 200–400 m, and serum tumor necrosis factor-α concentrations of at least 2.5 pg/mL [18]. Therefore, the trials evaluated clinically distinct populations with different degrees of frailty, functional impairment, and inflammatory enrichment, limiting their direct comparability and the generalizability of the findings across the full frailty spectrum. Furthermore, none of the trials required a diagnosis of sarcopenia according to internationally accepted criteria or confirmed low muscle quantity or quality using imaging-based measurements [4] Consequently, the available randomized evidence relates primarily to aging frailty or prefrailty and should not be interpreted as direct evidence of efficacy for sarcopenia.

**Figure 2:**
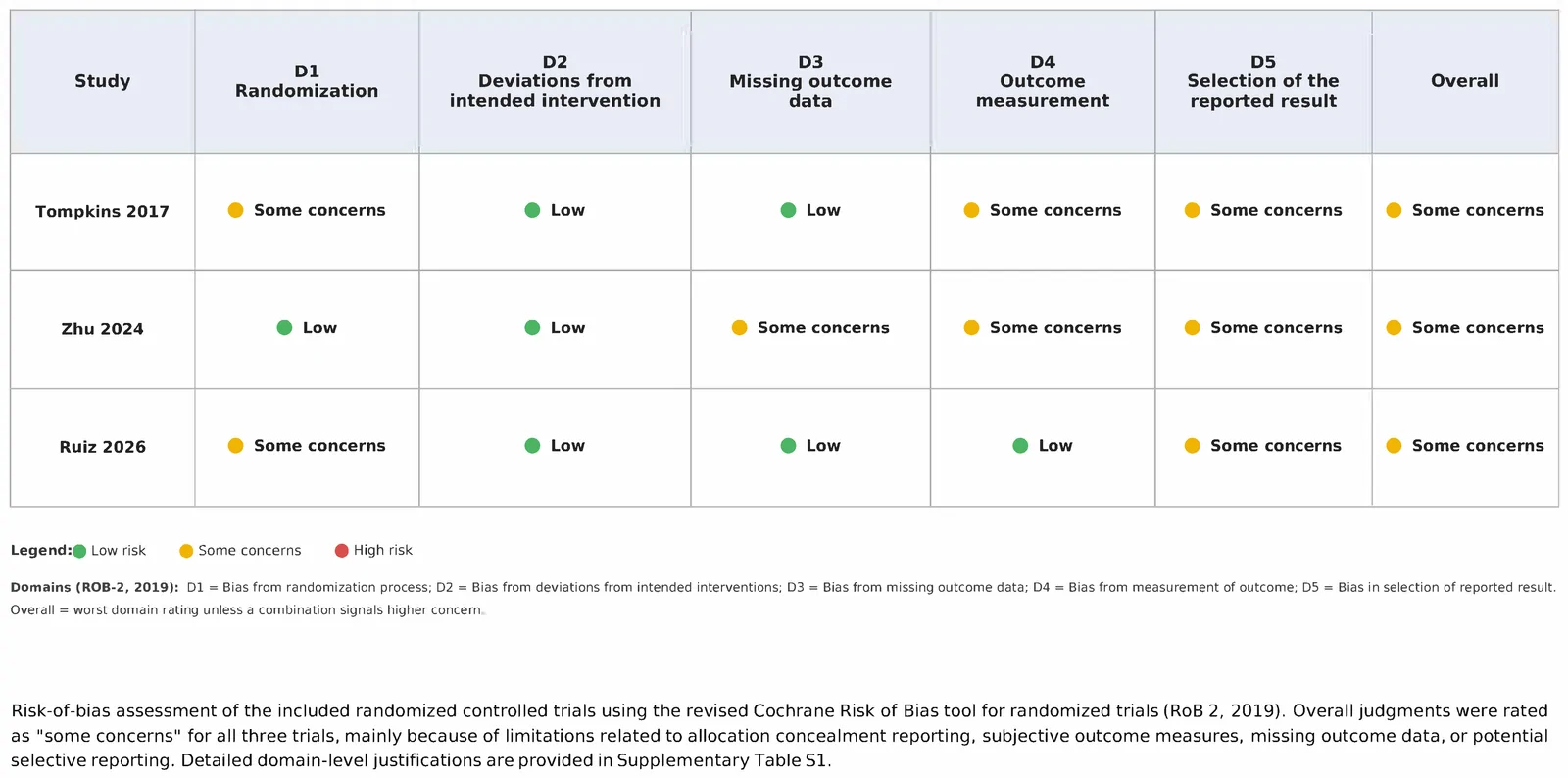
The Revised Cochrane Risk of Bias Tool for Randomized Trials [24] for assessing the methodological quality of the included randomized controlled trials.

Patient and study characteristics are presented in Table 1, whereas safety and efficacy outcomes are summarized in Table 2. Risk-of-bias judgments are summarized in Figure 2, while detailed domain-level justifications are provided in Supplementary Table S1.

### 3.2. Efficacy Outcomes

Improvements in functional performance were reported across all included trials; however, the magnitude, consistency, and statistical robustness of these effects varied substantially between studies.

In the phase II randomized controlled trial by Tompkins et al. [19], participants receiving 100 million allogeneic bone-marrow-derived MSCs demonstrated a significant improvement in 6MWT distance at six months compared with baseline (345.9 ± 103.4 m vs. 410.7 ± 155.4 m; *p* = 0.011), whereas no significant improvement was observed in either the 200-million cell group or the placebo group. Similarly, short physical performance battery (SPPB) scores improved significantly only in the 100-million cell group (median 10.5 vs. 12.0; *p* = 0.031). Pulmonary function, assessed by forced expiratory volume in 1 s (FEV1), also improved in the lower-dose group (2.5 ± 0.66 L vs. 2.6 ± 0.77 L; *p* = 0.025). In contrast, no significant differences were observed in handgrip strength, gait speed, fatigue indices, or cardiac function measures. These findings suggest a potentially dose-sensitive and non-linear therapeutic response; however, the limited sample size and exploratory design precluded definitive dose–response conclusions [19].

In the study by Zhu et al. [20], quality of life (QoL), assessed using the 36-Item Short Form Health Survey (SF-36) physical component score, was defined as the primary endpoint. Within the MSC group, SF-36 physical component scores improved from the first post-treatment assessment and remained significantly higher than baseline throughout follow-up. At six months, the change from baseline was significantly greater in the MSC group than in the placebo group (*p* = 0.042) [20]. Similarly, EuroQol visual analogue scale (EQ-VAS) scores improved significantly from month 2 onward (*p* = 0.023), with sustained improvement at six months (*p* = 0.002) [20]. Functional performance measures also favored MSC therapy. TUG performance showed a repeated between-group difference over follow-up (reported *p* < 0.05), while grip strength at six months was greater in the MSC group than in the placebo group (25.44 ± 5.44 kg versus 18.33 ± 10.11 kg; *p* = 0.002). At six months, the mean time required to complete the 4MWT was 4.04 ± 0.45 s in the MSC group and 6.09 ± 3.39 s in the placebo group (reported *p* = 0.021). However, the corresponding changes from baseline were −1.00 s and +0.19 s, respectively; therefore, the 2.05 s value represents the cross-sectional difference between groups at six months rather than a placebo-adjusted difference in change from baseline [20].

Furthermore, significant reductions in circulating inflammatory cytokines were observed, including tumor necrosis factor-alpha (TNF-α; *p* = 0.034) and interleukin-17 (IL-17; *p* = 0.033). Nevertheless, the relatively small sample size and variability across endpoints limit the generalizability and statistical strength of these findings [20].

The most robust efficacy evidence was provided by th e phase 2b dose-escalation trial by Ruiz et al. [18], in which functional performance was primarily evaluated using the 6MWT as the predefined primary endpoint. A significant dose–response relationship was identified, with increasing MSC doses associated with progressively greater functional improvement. At nine months, the placebo-adjusted difference in change from baseline in 6MWT distance was 63.4 m for the 200-million-cell group (95% CI: 17.1–109.6 m; *p* = 0.0077) and 49.2 m for the 50-million-cell group (95% CI: 10.9–87.5 m; *p* = 0.0122) [18]. Although the improvement in 6MWT appears clinically encouraging, its clinical significance should be interpreted with caution, given the absence of consistent improvements across secondary functional outcomes and the exploratory nature of the study. At six months, the highest-dose group demonstrated a 41.3 m improvement versus placebo, although this did not reach statistical significance (*p* = 0.0635). The larger between-group separation observed at nine months reflected both sustained improvement in selected active-treatment groups and a decline in the placebo group, as detailed below [18]. However, not all secondary functional endpoints demonstrated statistically significant improvements. Specifically, PROMIS Physical Function, PROMIS Mobility, and PROMIS Upper Extremity scores did not significantly differ from placebo, despite moderate correlations between PROMIS Physical Function and changes in 6MWT performance (r = 0.31–0.44). These findings suggest that MSC therapy may exert a more pronounced effect on endurance-related mobility and functional capacity rather than on maximal strength or short-duration performance outcomes [18].

Sample-size planning differed substantially across the three trials. Tompkins et al. enrolled 10 participants per group and explicitly reported that no formal statistical justification had been performed because the primary objective was safety; the efficacy analyses were exploratory, and no adjustment for multiple comparisons was applied [19]. The investigators subsequently estimated that approximately 30 participants per group would have been required to provide adequate power for the observed between-group difference in 6MWT distance. Zhu et al. calculated a sample size of 15 participants per group using a two-sided α of 0.05 and 80% power, based on an earlier MSC trial; however, the numerical effect size, the target between-group difference, and any MCID used in this calculation were not reported [20]. Consequently, it is not possible to determine whether that study was specifically powered to detect a clinically meaningful difference in its primary SF-36 physical component outcome or in its secondary functional outcomes. In contrast, Ruiz et al. calculated that 30 participants per group would provide approximately 80% power, using a one-sided α of 0.025, to detect a placebo-adjusted 56 m difference in 6MWT distance, corresponding to an effect size of 0.75 and an assumed common standard deviation of 75 m [18]. This target exceeded both the 17.8 m MCID estimated in moderately frail older adults and the approximately 20 m and 50 m thresholds proposed for small and substantial meaningful changes, respectively, in older populations [26,27]. Nevertheless, the 200-million-cell group, which was introduced after enrollment had begun, included only 16 participants and therefore did not reach the planned sample size. Ruiz et al. also prespecified a 2-point MCID for the PROMIS Physical Function SF20, although no statistically significant treatment–placebo difference was demonstrated for this outcome [18].

The trajectory of the placebo group in the Ruiz et al. trial was not uniform across follow-up. The least-squares mean change in 6MWT distance in the placebo group was +16.1 m at three months (95% CI: −3.5 to 35.6 m) and +8.0 m at six months (95% CI: −17.6 to 33.7 m), followed by a decline of −15.5 m at nine months (95% CI: −43.3 to 12.3 m); none of these within-group changes was statistically significant [18]. Accordingly, the 41.3 m treatment–placebo difference observed with 200 million cells at six months reflected a 49.3 m improvement in the active-treatment group and an 8.0 m improvement in the placebo group. At nine months, the larger 63.4 m difference reflected both a sustained 47.9 m improvement in the active-treatment group and a 15.5 m decline in the placebo group [18]. Thus, the increasing separation between groups over time cannot be attributed exclusively to progressive improvement in treated participants and should also be interpreted in relation to the changing placebo trajectory.

### 3.3. Quality of Life and Inflammatory and Immune Biomarkers

Improvements in health-related QoL were observed across the included trials, particularly in domains related to physical function. In the study by Zhu et al. [20], these improvements were evident alongside measurable gains in functional performance.

In addition, Zhu et al. [20] reported significant reductions in circulating tumor necrosis factor-alpha (TNF-α; *p* = 0.034) and interleukin-17 (IL-17; *p* = 0.033) concentrations at six months following HUC-MSC administration compared with placebo. In contrast, no significant between-group differences were observed in interleukin-8 (IL-8) or interferon-gamma (IFN-γ) concentrations. These findings suggest that the inflammatory response was not uniform across all measured cytokines, but was mainly reflected in changes in TNF-α and IL-17.

Tompkins et al. [19] provided further evidence of immune modulation at the cellular level. The proportion of early activated CD3+ CD69+ T cells decreased significantly in the 200-million-cell group at six months (*p* = 0.004), whereas no significant reduction was observed in the 100-million-cell or placebo groups. Late activated CD3+ CD25+ T cells decreased in both the 100-million-cell and 200-million-cell groups (*p* = 0.007 and *p* = 0.048, respectively). In addition, the proportion of CD8+ T cells decreased in the 200-million-cell group (*p* = 0.022), accompanied by an increase in the CD4/CD8 ratio (*p* = 0.014). No significant change in the proportion of CD4+ T cells was observed.

Changes in TNF-α were also observed at both the circulating and cellular levels. Serum TNF-α concentrations decreased significantly in the 100-million-cell group at six months (*p* = 0.031), but not in the 200-million-cell or placebo groups. The proportion of B cells expressing intracellular TNF-α decreased in both MSC-treated groups (*p* < 0.0001 for the 100-million-cell group and *p* = 0.002 for the 200-million-cell group), with no significant change in the placebo group. No significant changes were observed in IL-6, C-reactive protein, D-dimer, fibrinogen, or complete blood cell counts. Thus, the immune response observed in this trial involved changes in both circulating inflammatory mediators and immune cell activation [19].

In the study by Ruiz et al. [18], patient-reported outcomes assessed using the PROMIS Physical Function scale demonstrated moderate correlations with improvements in 6MWT (r: 0.3–0.44). These findings suggest that objective gains in mobility are partially reflected in patient-perceived functional status, although the strength of this association remains moderate, indicating that subjective and objective measures capture overlapping but distinct dimensions of functional improvement.

### 3.4. Safety Outcomes

Across all three RCTs, intravenous MSC administration was well-tolerated. No treatment-related serious adverse events were reported during the predefined safety assessment periods. In all studies [18,19,20], the incidence of adverse events was comparable between MSC-treated and placebo groups, and the reported events were generally mild to moderate and transient in nature. No clinically significant immunological reactions were reported during follow-up. However, the combined sample of 208 participants and the follow-up periods of six to twelve months were insufficient to exclude uncommon, delayed, immunological, thrombotic, or product-related adverse effects. Therefore, the available findings should be interpreted as preliminary evidence of short-term tolerability rather than confirmation of an established favorable long-term safety profile [18,19,20].

### 3.5. Vascular and Mechanistic Findings

Ruiz et al. [18] assessed a panel of eight circulating biomarkers related to vascular function and angiogenesis, together with C-reactive protein, to identify a potential biomarker of laromestrocel activity. Among the biomarkers examined, soluble TIE2 (sTIE2) showed the clearest treatment-related and dose-dependent response. At six months, circulating sTIE2 concentrations decreased significantly in the 100-million-cell group compared with placebo, with a placebo-adjusted difference of −400.0 pg/mL (95% CI: −762.58 to −37.51 pg/mL; *p* = 0.021). The reduction remained significant in the 100-million-cell group at nine months (*p* = 0.001), while a significant decrease was also observed in the 200-million-cell group at the same time point. Dose–response analysis further supported an association between increasing laromestrocel doses and decreasing sTIE2 concentrations.

TIE2, also known as TEK, is a receptor tyrosine kinase expressed mainly on microvascular endothelial cells and endothelial progenitor cells. It is activated by the angiopoietins ANGPT1 and ANGPT2 and participates in the regulation of vascular stability, angiogenesis, and inflammatory responses. The extracellular domain of membrane-bound TIE2 may undergo proteolytic cleavage, particularly through matrix metalloproteinase-14, resulting in the release of sTIE2 into the circulation. Increased circulating sTIE2 may therefore reflect enhanced TIE2 cleavage and impaired endothelial homeostasis.

Laromestrocel-conditioned medium was also shown to contain tissue inhibitor of metalloproteinase-2 (TIMP2), as well as angiogenic factors, including vascular endothelial growth factor-A and placental growth factor. Since TIMP2 inhibits matrix metalloproteinase activity, its secretion by laromestrocel provides a biologically plausible link between MSC paracrine activity and the reduction in circulating sTIE2. Nevertheless, sTIE2 was assessed as an exploratory biomarker, and endothelial function was not directly measured. Therefore, the observed reduction in sTIE2 should be considered preliminary evidence of a possible vascular effect rather than definitive proof of the underlying mechanism [18].

### 3.6. Quantitative Synthesis

Because only three trials were eligible and they differed substantially in participant eligibility criteria, MSC products and dosing regimens, primary outcomes, and follow-up time points, statistical pooling was not considered methodologically appropriate. Accordingly, no pooled effect estimate or statistical measure of between-study heterogeneity was calculated, and the findings were synthesized narratively.

## 4. Discussion

Previous reviews have addressed the biological rationale and potential therapeutic use of MSCs in sarcopenia and related musculoskeletal aging syndromes [21,22,23]. The present review differs by restricting its evidence base to randomized controlled trials of intravenous MSC administration in clinically characterized older adults with age-related frailty and by integrating functional, safety, cell-product, and biomarker outcomes. Only three early-phase randomized trials were eligible, and none specifically enrolled participants with consensus-defined sarcopenia [18,19,20]. Although these studies provide preliminary signals of biological activity and a possible functional benefit, their small or uneven group sizes, exploratory analyses, heterogeneous populations and cell products, and lack of independent replication preclude firm conclusions regarding clinical efficacy.

The RoB 2 assessment resulted in an overall judgment of “some concerns” for all three trials, reflecting one or more limitations related to allocation concealment reporting, missing outcome data, reliance on subjective patient-reported outcomes, or potential selective reporting (Figure 2). These methodological concerns, together with the small or uneven group sizes and the multiplicity of exploratory analyses, further limit confidence in the reported efficacy estimates.

Frailty should not be conflated with the physiological changes expected during normal ageing. Although chronological age is associated with a progressive population-level decline in physiological function, neither advanced age alone nor the increasing prevalence of frailty in older age groups is sufficient to establish clinically meaningful frailty [1,6]. No universally accepted age-adjusted threshold separates normal physiological ageing from frailty. In clinical practice and research, frailty becomes clinically meaningful when a validated assessment identifies a loss of physiological reserve that is associated with disproportionate vulnerability to stressors, functional deterioration, and an increased risk of adverse outcomes compared with individuals of a similar chronological age [1,4,17]. This distinction is particularly important in the present review because the included trials enrolled populations across different levels of vulnerability, prefrailty, and established clinical frailty. Consequently, treatment effects observed in one frailty subgroup cannot be assumed to apply across the entire severity spectrum. Moreover, because frailty, sarcopenia, and disability are related but distinct constructs, improvements in mobility-based outcomes should not be interpreted as evidence of reversal of sarcopenia or disability in the absence of condition-specific diagnostic assessments [4,5].

Across the three trials, the functional findings were heterogeneous rather than uniformly positive. Improvements were concentrated in selected mobility- and endurance-related outcomes, while treatment effects were not reproduced consistently across cell doses, follow-up time points, or complementary functional and patient-reported measures [18,19,20]. The trials also contributed unequally to the evidence base: Tompkins et al. and Zhu et al. were small exploratory studies, whereas the phase 2b trial by Ruiz et al. enrolled a larger overall sample and incorporated a prespecified dose–response analysis. Nevertheless, the 200-million-cell group included only 16 participants, the pairwise comparison with placebo at the prespecified six-month primary time point was inconclusive, and the later effect estimates remained imprecise [18]. Accordingly, the available evidence supports a preliminary efficacy signal, principally for endurance-related mobility, but not a consistent or confirmatory treatment effect.

The distinction between statistical significance and clinical importance materially affects the interpretation of these findings. In the Tompkins et al. trial, the 100-million-cell group exhibited a within-group increase of 64.8 m in 6MWT distance and a 1.5-point increase in the median SPPB score [19]. These magnitudes exceed the approximately 50 m and 1.0-point thresholds proposed for substantial meaningful changes in 6MWT and SPPB performance, respectively [27]. However, these were within-group changes derived from an exploratory study with only 10 participants per arm, no formal efficacy-based sample-size calculation, and no adjustment for multiple comparisons. Because an adequately powered treatment–placebo comparison was not demonstrated, these changes cannot be considered definitive evidence of a clinically meaningful treatment effect. Similarly, Zhu et al. reported a significantly greater change from baseline in the SF-36 physical component score in the MSC group than in the placebo group at six months (*p* = 0.042); however, the trial did not report the numerical between-group treatment effect, its 95% confidence interval, or an MCID applicable to the scoring approach used [20]. Furthermore, the reported physical component scores exceeded 200 points, indicating an aggregated scoring approach rather than the conventional norm-based SF-36 summary score. Published MCIDs for the standard SF-36 physical component score therefore cannot be directly applied without clarification of the scoring algorithm.

Ruiz et al. provided the most explicit evaluation of clinical relevance because the trial was prospectively powered to detect a 56 m placebo-adjusted difference in 6MWT distance [18]. At the prespecified six-month primary time point, the overall dose–response relationship was statistically significant (*p* = 0.0321), but the pairwise difference between the 200-million-cell group and placebo was 41.3 m (95% CI: −2.4 to 84.9 m; *p* = 0.0635) and was therefore inconclusive. At nine months, the corresponding difference reached 63.4 m (95% CI: 17.1–109.6 m; *p* = 0.0077), exceeding the approximately 50 m benchmark for a substantial meaningful change [27]. Nevertheless, the confidence interval remained wide and included values below the frailty-specific MCID of 17.8 m, while the highest-dose group included only 16 participants rather than the 30 planned in the sample-size calculation [18,26]. The 49.2 m placebo-adjusted difference observed with 50 million cells at nine months was also accompanied by a wide 95% confidence interval (10.9–87.5 m) [18]. Thus, the available results support the possibility of a clinically important benefit, particularly for endurance-related mobility, but do not yet establish its magnitude with sufficient precision or high certainty.

An important observation is the temporal pattern of response. In the Ruiz et al. study, the between-group separation in 6MWT distance was greater at nine months than at six months. However, this later separation reflected both improvement in selected laromestrocel groups and a decline in the placebo group and therefore cannot, by itself, establish a delayed therapeutic mechanism [18]. A delayed biological effect remains plausible in view of the proposed paracrine and immunomodulatory actions of MSCs, but this interpretation remains hypothesis-generating and requires confirmation in adequately powered trials with prespecified repeated assessments [18,20,28]. MSCs may modulate chronic low-grade inflammation by reducing the production of pro-inflammatory cytokines and altering immune-cell activation, thereby attenuating inflammaging processes associated with frailty [28]. In addition, MSCs may improve endothelial and vascular function through the secretion of angiogenic and trophic factors, promoting vascular homeostasis and tissue perfusion [14,29]. Experimental evidence also suggests that MSC therapy may enhance skeletal muscle regeneration and support cellular homeostasis through paracrine and trophic mechanisms, potentially contributing to improvements in endurance-related physical performance observed in frail individuals [14,28,30].

Placebo responses and other non-specific effects should also be considered when interpreting the functional findings. All three trials were described as double-blind and placebo-controlled [18,19,20]. Zhu et al. and Ruiz et al. explicitly reported that the placebo was matched to, or visually indistinguishable from, the active infusion [18,20]. In the report by Tompkins et al., however, the composition and appearance of the placebo were not described in sufficient detail to independently evaluate the adequacy of placebo matching [19].

Regarding concomitant interventions, the published reports by Tompkins et al. and Ruiz et al. did not describe whether physiotherapy, structured exercise, nutritional supplementation, or rehabilitation undertaken during follow-up was prohibited, standardized, or prospectively recorded [18,19]. Zhu et al. instructed participants not to change their lifestyles during the intervention period, but adherence to this instruction was not objectively assessed, and detailed longitudinal information on exercise, dietary intake, rehabilitation, or other concomitant interventions was not reported [20]. The absence of such reporting does not demonstrate that co-interventions occurred, but it prevents confirmation that potentially effective non-pharmacological treatments remained balanced between groups, particularly in trials with small sample sizes.

The transient early improvement in 6MWT distance among placebo-treated participants in the Ruiz et al. trial may reflect natural within-person variability, regression to the mean, increased motivation associated with trial participation, or a practice effect from repeated functional testing [18,31]. Ruiz et al. attempted to improve baseline reproducibility by requiring two screening 6MWTs to be within 15% of each other, which may have reduced, but cannot completely exclude, familiarization effects [18]. Repeated contact with research personnel and awareness of being observed may also alter health-related behavior, although the existence and magnitude of a Hawthorne effect cannot be reliably quantified in the present trials [32]. Therefore, within-group improvement alone should not be interpreted as evidence of treatment efficacy; causal interpretation should be based primarily on prospectively defined between-group differences. Future trials should standardize or stratify access to exercise, physiotherapy, nutritional support, and rehabilitation; prospectively record changes in medications and co-interventions; incorporate standardized test familiarization; and consider objective physical-activity monitoring throughout follow-up.

The biomarker findings provide preliminary evidence of biological activity but do not demonstrate a consistent molecular response across trials. Tompkins et al. reported reductions in selected indices of T-cell activation and B-cell intracellular TNF-α, whereas serum TNF-α decreased only in the 100-million-cell group [19]. Zhu et al. similarly observed reductions in circulating TNF-α and IL-17, but not in IL-8 or IFN-γ [20]. Because these effects were selective, lacked a consistent dose–response pattern, and were not independently replicated, they should be regarded as exploratory signals of immunomodulation rather than evidence of a confirmed anti-inflammatory mechanism [19,20,33].

Ruiz et al. reported a dose-dependent reduction in circulating sTIE2, providing a possible link between laromestrocel administration and angiopoietin–TIE2 signaling [18,34,35]. Although the secretion of TIMP2 and angiogenic factors by laromestrocel provides a biologically plausible paracrine mechanism, the trial did not directly assess endothelial function or demonstrate a patient-level association between changes in sTIE2 and 6MWT performance [18]. Therefore, sTIE2 should be considered a candidate biomarker of biological activity rather than a validated mediator of clinical benefit. Overall, the findings support the biological plausibility of immunomodulatory and vascular effects but do not establish a definitive mechanism of action [18,19,20].

The biological heterogeneity of the administered MSC products should also be considered. Tompkins et al. [19] and Ruiz et al. [18] administered allogeneic bone-marrow-derived MSC products, whereas Zhu et al. [20] used fifth-passage human umbilical-cord-derived MSCs. The studies also differed in total cell dose, number of administrations, manufacturing procedures, culture conditions, cell viability criteria, and potency assessment. Such factors may influence the secretory profile, immunomodulatory properties, angiogenic activity, and clinical potency of the final MSC product. Therefore, differences in functional and biomarker responses across the included trials cannot be attributed solely to cell dose and may also reflect differences in MSC source and manufacturing [18,19,20,36].

Although sarcopenia represents a major biological component of frailty, no RCTs specifically targeting sarcopenia with MSC therapy were identified in the present review. Nevertheless, improvements in muscle-related functional outcomes, including endurance and physical performance measures, suggest that MSC therapy may exert indirect beneficial effects on skeletal muscle physiology through paracrine mechanisms rather than through direct myogenic regeneration [18]. Experimental and preclinical studies further support this hypothesis, demonstrating that MSCs may promote muscle regeneration, reduce chronic inflammation, and improve the regenerative capacity of skeletal muscle tissue through paracrine and trophic mechanisms [14,28,30].

However, the included clinical trials mainly assessed physical performance rather than direct measures of skeletal muscle biology. None of the studies evaluated changes in muscle mass using imaging, muscle histology, myogenic differentiation, mitochondrial function within skeletal muscle, or molecular markers of muscle protein synthesis and degradation. Therefore, improvements in mobility and endurance cannot be interpreted as direct evidence of myogenic regeneration or as proof of a disease-modifying effect on sarcopenia. Any potential effect on skeletal muscle may be indirect and mediated through reduced systemic inflammation, improved vascular function, enhanced tissue perfusion, or broader restoration of tissue homeostasis.

At present, the management of sarcopenia remains largely centered on exercise and nutritional interventions [37]. In particular, protein supplementation combined with vitamin D has demonstrated beneficial effects on muscle strength in older adults with sarcopenia. In a systematic review and meta-analysis by Gkekas et al., vitamin D plus protein supplementation improved handgrip strength [standard mean difference (SMD) 0.38; 95% CI 0.01–0.75; *p* = 0.04] and sit-to-stand performance (SMD 0.25; 95% CI 0.06–0.43; *p* = 0.007), although no consistent effect on muscle mass or walking speed was observed [38]. These findings suggest that multimodal therapeutic approaches targeting inflammation, nutrition, and physical performance may be required for optimal management of frailty-related sarcopenia [38]. Given their immunomodulatory and regenerative properties, MSC-based therapies may represent a promising future therapeutic strategy, either alone or in combination with established nutritional and exercise interventions. Nevertheless, MSC therapy should currently be considered an investigational approach, and its role as an adjunct to established interventions requires evaluation in adequately powered clinical trials.

The clinical value of MSC therapy must ultimately be evaluated within a formal risk–benefit framework. No treatment-related serious adverse events were identified across the three included trials, providing preliminary evidence of short-term tolerability [18,19,20]. However, this safety evidence was derived from only 208 participants, with individual trial sample sizes ranging from 30 to 148 and follow-up periods of six to twelve months. These data are insufficient to exclude uncommon, delayed, immunological, thrombotic, or product-related adverse effects. Moreover, none of the trials demonstrated reductions in hard clinical outcomes such as falls, fractures, hospitalization, long-term care admission, disability progression, or mortality. MSC administration also requires donor screening, good manufacturing practice-compliant cell expansion, product characterization and potency assessment, cryopreservation, transport, specialized clinical administration, and post-infusion monitoring [18,19,20,36]. Therefore, the absence of an evident short-term safety signal should be interpreted as preliminary tolerability rather than as confirmation of a favorable long-term risk–benefit profile. None of the included RCTs reported intervention costs, healthcare-resource utilization, quality-adjusted life-years, or incremental cost-effectiveness ratios; consequently, no conclusion can currently be drawn regarding the cost-effectiveness of intravenous MSC therapy for aging frailty [18,19,20]. This represents an important evidence gap because frailty is associated with substantially greater healthcare expenditure than either prefrailty or robustness [39]. Current international guidance recommends multicomponent physical activity incorporating resistance training as first-line treatment for physical frailty, together with protein or caloric supplementation when weight loss or undernutrition is present and a comprehensive care plan addressing reversible contributors to frailty [40]. Economic evaluations of physical activity interventions in older adults are substantially more developed than those of MSC therapy: a scoping review found that most evaluated programs were more effective but more costly than no intervention, while a smaller proportion were both more effective and cost-saving, although results remained dependent on the intervention and healthcare setting [41]. Future MSC trials should therefore evaluate cell therapy as an adjunct to optimized exercise, nutritional, and multidisciplinary care rather than solely against placebo. They should prospectively collect manufacturing and administration costs, adverse-event costs, healthcare utilization, informal-care requirements, quality-adjusted life-years, and longer-term outcomes, allowing calculation of incremental cost-effectiveness from both healthcare-system and societal perspectives.

Despite these encouraging findings, several limitations should be acknowledged. First, the number of available studies was small, with limited sample sizes, reducing their statistical power and limiting the generalizability of the findings. Second, considerable heterogeneity was observed in terms of MSC source, manufacturing procedures, dosing regimens, number of administrations, follow-up duration, and outcome measures, which complicates comparisons and limits the identification of optimal treatment strategies. The small number of eligible trials and their substantial clinical and methodological heterogeneity precluded a methodologically defensible meta-analysis; consequently, no pooled effect estimate could be calculated, and between-study heterogeneity and publication bias could not be meaningfully assessed.

Third, all included studies represent early-phase clinical trials, and therefore long-term efficacy and durability of MSC therapy remain uncertain. Fourth, the biomarker analyses were exploratory and were not standardized across studies. Each trial assessed a different set of inflammatory, immune, or vascular markers, and none of the principal biomarker findings was independently replicated in another RCT. In addition, the available trials did not establish patient-level relationships between changes in biological markers and improvements in functional outcomes. Fifth, no trial directly assessed skeletal muscle structure or molecular pathways relevant to sarcopenia. Finally, the absence of hard clinical endpoints and sufficiently long follow-up limits assessment of the durability and broader clinical consequences of treatment.

Future research should now move beyond small exploratory studies toward adequately powered, multicenter, confirmatory randomized controlled trials. Eligibility should require a prespecified, validated definition of frailty and a clearly defined severity category, rather than chronological age or functional limitation alone [4,17]. Frailty, sarcopenia, and disability should be assessed and reported separately, because they represent related but non-equivalent clinical constructs. Trials specifically targeting sarcopenia should apply consensus diagnostic criteria incorporating muscle strength, muscle quantity or quality, and physical performance [4]. Randomization should be stratified by study center and major prognostic variables, including baseline frailty severity and physical performance, with age- and sex-related subgroup analyses prespecified and adequately powered.

Sample-size calculations should be based on a prespecified between-group difference that is clinically meaningful, rather than merely statistically detectable, and should account for expected attrition. For the 6MWT and other functional outcomes, the target effect should be justified against an applicable MCID, while recognizing that existing thresholds may not be directly transferable across frailty severity levels or populations [26,27]. A single primary outcome and primary assessment time point should be defined prospectively. If the 6MWT is retained, its administration should be rigorously standardized, with consideration of a familiarization or repeat baseline assessment to limit learning effects [31]. Treatment effects should be presented as between-group estimates with 95% confidence intervals and, where appropriate, supplemented by responder analyses based on clinically meaningful thresholds. Secondary outcomes should follow a prespecified hierarchy and include complementary measures of mobility, strength, frailty status, patient-reported physical function, and quality of life, together with clinically consequential endpoints such as falls, hospitalization, progression of disability, institutionalization, and mortality. Analyses should follow the intention-to-treat principle, with prespecified methods for addressing missing data, intercurrent events, and multiplicity.

Methodological safeguards are also required to minimize bias and non-specific treatment effects. Future trials should use concealed allocation, an indistinguishable placebo, and blinding of participants, treating personnel, outcome assessors, and statisticians wherever feasible. Outcome assessors should undergo centralized training, and functional testing procedures should be harmonized across centers. Concomitant exercise, physiotherapy, nutritional supplementation, and rehabilitation should either be standardized between study groups or prospectively documented, with adherence monitored using predefined methods. This is essential for distinguishing a treatment-specific effect from learning, placebo, Hawthorne, or co-intervention effects [31,32,40]. Follow-up should extend beyond the 6–12-month periods used in the available trials to establish durability and identify delayed or uncommon adverse events, supported by independent safety monitoring.

The cellular intervention itself should be defined as rigorously as the clinical population. Cell source, donor eligibility, passage number, culture and expansion conditions, cryopreservation and thawing procedures, viability, dose, administration schedule, release criteria, and batch-specific potency should be standardized and transparently reported [25,36]. Mechanistic analyses should use a limited set of prospectively defined biomarkers collected at standardized time points, with patient-level analyses examining whether biological changes precede or correlate with clinical improvement. Trials targeting sarcopenia should additionally include objective assessment of muscle quantity using dual-energy X-ray absorptiometry or bioelectrical impedance analysis and, where feasible, imaging-based assessment of muscle quantity and quality using computed tomography or magnetic resonance imaging, alongside validated measures of muscle strength and physical performance [26]. An embedded economic evaluation should assess resource use, costs, quality-adjusted life-years, and incremental cost-effectiveness relative to optimized exercise, nutritional, and multidisciplinary care [39,40,41]. Protocols should be prospectively registered and developed in accordance with SPIRIT 2025 [42], and final reports should follow CONSORT 2025 [43].

## 5. Conclusions

Current randomized evidence provides preliminary evidence of short-term tolerability and hypothesis-generating signals of possible benefit in selected mobility-related functional outcomes, quality of life, and inflammatory, immune, or vascular biomarkers. However, only three heterogeneous early-phase trials were available, with small or uneven group sizes, imprecise effect estimates, methodological concerns, and no direct evidence in participants with consensus-defined sarcopenia. Consequently, efficacy, durability, and long-term risk–benefit remain unestablished. The biomarker findings are exploratory and do not establish a definitive mechanism of action. Adequately powered, multicenter confirmatory trials with validated population definitions, MCID-based endpoints, standardized co-interventions, longer follow-up, and prospective economic evaluation are required.

## Figures and Tables

**Table 1:** Main characteristics of the clinical studies included in the systematic review.

Study (Author, Year)	Study Design	MSC Source	Sample Size (MSC/Control)	Mean Age (Years)	Dose	Follow-Up Duration	Main Outcomes Assessed
Tompkins et al., 2017 [19]	Phase I/II dose-escalation trial	Bone marrow–derived allogeneic MSCs	30 (three arms: 100 M, 200 M MSC and placebo)	75.5 ± 7.3	Single IV infusion; 100 or 200 million cells	12 months	Safety, 6-min walk test, SPPB, pulmonary function, inflammatory markers
Zhu et al., 2024 [20]	Phase I/II randomized double-blind placebo-controlled trial	Umbilical cord–derived MSCs	30 (15 MSC/15 placebo)	67 ± 6	Two IV infusions; 1 × 10^6^ cells/kg administered 1 month apart	6 months	Quality of life (SF-36 PCS, primary endpoint), gait speed, grip strength, timed up-and-go, inflammatory markers
Ruiz et al., 2026 [18]	Phase 2b randomized, double-blind, placebo-controlled, dose-escalation trial	Bone marrow–derived allogeneic MSCs (laromestrocel)	148 (multiple dose groups vs. placebo)	74.3–76.8	Single IV infusion; 25, 50, 100, or 200 M cells	9 months	6-min walk test (primary endpoint), PROMIS physical function, mobility measures, inflammatory and vascular biomarkers (including sTIE2)

**Abbreviations:** IV: intravenous; M: million; MSC: mesenchymal stem cells; PCS: Physical Component Summary; PROMIS: Patient-Reported Outcomes Measurement Information System; SF-36: 36-Item Short Form Health Survey; sTIE2: soluble tyrosine kinase with immunoglobulin-like and EGF-like domains 2; vs.: versus.

**Table 2:** Summary of safety and efficacy outcomes reported in the included studies.

Study (Author/ Year)	Safety Outcomes	Physical Performance Outcomes	Quality of Life Outcomes	Inflammatory Markers
Tompkins et al., 2017 [19]	No treatment-related serious adverse events; one unrelated death in the 200-million-cell group during follow-up	+22.6 m (3 months) and +39.3 m (6 months) in 6-min walk test (100 million-cell dose)	Not assessed	Decrease in TNF-α at 6 months
Ruiz et al., 2026 [18]	No treatment-related serious adverse events; adverse event rates comparable across groups	Dose-dependent improvement in 6MWT; +63.4 m at 9 months in the 200M group vs. placebo (*p* = 0.0077); improvement in Clinical Frailty Scale (CFS) status	Improvement in PROMIS Physical Function scores was associated with increased 6MWT distance	Dose-dependent reduction in sTIE2 levels
Zhu et al., 2024 [20]	No serious adverse events reported	Improvement in timed up-and-go, grip strength and 4-m walking speed	Improvement in SF-36 Physical Component Summary and EQ-VAS scores compared with placebo	Decrease in TNF-α and IL-17

**Abbreviations:** CFS: Clinical Frailty Scale; FEV: forced expiratory volume; IL-17: interleukin-17; m: meters; PROMIS: Patient-Reported Outcomes Measurement Information System; SPPB: short physical performance battery; sTIE2: soluble tyrosine kinase with immunoglobulin-like and EGF-like domains 2; TNF-α: tumor necrosis factor α.

## Data Availability

No new data were generated in this study. All data supporting the findings of this systematic review are available in the cited publications.

## Supplementary Materials

The following supporting information can be downloaded at https://www.mdpi.com/article/10.3390/ijms27177596/s1.
