# Supplementary material for: Safety and Efficacy of Mesenchymal Stem Cell Therapy in Aging Frailty: A Systematic Review
Source: Int J Mol Sci. 2026 Aug 25;27(17):7596. doi: 10.3390/ijms27177596 (PMC13566211; doi:10.3390/ijms27177596)
Supplement: Supplementary file 1 [file ijms-27-07596-s001.zip › ijms-4495718-supplementary.pdf]

Supplementary Table S1. Detailed RoB 2 domain-level judgments and justifications

The judgments and detailed justifications below reproduce the content of the original Figure 2. No additional interpretation has been introduced.

Study	RoB 2 domain	Judgment	Detailed justification
**Tompkins 2017**<br>Phase II RCT · BM-MSC<br>n=30 · 6-mo follow-up	D1 Randomization	Some concern	1:1:1 block randomization reported; allocation concealment method not explicitly described. No formal sample size justification stated.
	D2 Deviations from intended intervention	Low	Double-blind; placebo-controlled. All 30 infused; no protocol deviations reported. Cardiopulmonary monitoring post-infusion. No evidence of unblinding.
	D3 Missing outcome data	Low	100% completed 1-yr follow-up except 1 unrelated death (200M) and 1 unrelated stroke (placebo). ITT population maintained. Missing data minimal.
	D4 Outcome measurement	Some concern	Patient-reported outcomes (SQOL-F, IIEF) susceptible to subjective bias despite blinding. 6MWT/SPPB/FEV1 are objective but performance-based. Immune assays objective; blinded lab reported.
	D5 Selection of reported result	Some concern	No pre-registered analysis plan cited beyond ClinicalTrials.gov registration. Multiple secondary endpoints tested without multiplicity correction. Selective reporting possible.
	Overall	Some concern	
**Zhu 2024**<br>Phase I/II RCT · HUC-MSC<br>n=30 · 6-mo follow-up	D1 Randomization	Low	Block randomization (block size 6), external statistician, sealed opaque envelopes. Both clinicians and research assistants blinded. Baseline characteristics well balanced.
	D2 Deviations from intended intervention	Low	Double-blind; matching placebo (same volume saline). Products matched in size, packaging, appearance, texture. No protocol deviations noted. No unblinding events reported.
	D3 Missing outcome data	Some concern	1 MSC participant withdrew at 2 months (12/15 completed month-6). 7.77% of scheduled visits missed; ITT used; however, differential attrition at months 2&3 (12/15 MSC vs 12&15/15 placebo). Imputation method not described.
	D4 Outcome measurement	Some concern	PCS of SF-36 and EQ-VAS are patient-reported; susceptibility to response bias despite blinding. Physical performance tests (TUG, 4MWT, grip) objective and assessor-blinded. Cytokine assays objective.
	D5 Selection of reported result	Some concern	Sample size based on prior MSC frailty trial (Tompkins). Pre-specified primary endpoint (PCS-SF36). Multiplicity not corrected across numerous secondary endpoints. Selective emphasis on significant results in abstract possible.
	Overall	Some concern	

Study	RoB 2 domain	Judgment	Detailed justification
**Ruiz 2026**<br>Phase IIb RCT · Laromestrocel<br>n=148 · 9-mo follow-up	D1 Randomization	Some concern	Central randomization (IQVIA) with block size 4 for initial 4 arms. 200M arm added mid-study after 92 enrolled; randomization scheme changed to center-stratified to accommodate — introduces potential for imbalance. 200M arm smaller (n=16).
	D2 Deviations from intended intervention	Low	Double-blind; identically appearing infusion bags. Independent pharmacovigilance + NIA-appointed DSMB oversight. Two temporary infusion interruptions (25M arm) both completed. No protocol deviations attributed to product.
	D3 Missing outcome data	Low	95.8% trial completion rate (137/143 mITT). 5 withdrew by choice. 4 lost to follow-up, 2 deaths (unrelated/unrelated). mITT analysis pre-specified. MMRM handles intermittent missing data. COVID-19 cited for some missed visits.
	D4 Outcome measurement	Low	6MWT (primary) is objective performance-based, standardized. PROMIS is validated patient-reported but susceptible to expectation bias — partially mitigated by blinding. CFS scored by caregiver/clinician. sTIE2 assay objective and blinded.
	D5 Selection of reported result	Some concern	Pre-registered (NCT03169231); two-component primary endpoint pre-specified (individual dose vs placebo at 6 mo + dose-response). Primary at 6 mo not significant; month-9 results highlighted. Biomarker (sTIE2) was exploratory pre-specified. TNF-1α excluded post-hoc due to sample degradation.
	Overall	Some concern	

Summary from the original Figure 2: Overall judgments were rated as "some concerns" for all three trials, mainly because of limitations related to allocation concealment reporting, subjective outcome measures, missing outcome data, or potential selective reporting.

Legend: Low risk; Some concerns; High risk.

Domains (ROB-2, 2019): D1 = Bias from randomization process · D2 = Bias from deviations from intended interventions · D3 = Bias from missing outcome data · D4 = Bias from measurement of outcome · D5 = Bias in selection of reported result. Overall = worst domain rating unless a combination signals higher concern.
